# Clinical factors associated with impaired near stereoacuity in children and adolescents with intermittent exotropia

**DOI:** 10.3389/fnins.2026.1790302

**Published:** 2026-03-16

**Authors:** Yunzhi Zhao, Xin Cui, Wenli Lu, Ailin Chen, Yanzi Liu, Zhicheng Xu, Wenchao Lyu, Jiaxun Li, Mengdi Wang, Haomin Gou, Yang Qiao, Song Mao, Shengyuan Chen, Jian Cui, Ya Gao, Fang Gao, Yufei Wei, Xuefeng Shi

**Affiliations:** 1 Clinical College of Ophthalmology, Tianjin Medical University, Tianjin, China; 2 Tianjin Key Laboratory of Ophthalmology and Visual Science, Tianjin Eye Institute, Tianjin Eye Hospital, Tianjin, China; 3 School of Medicine, Nankai University, Tianjin, China; 4 School of Public Health, Tianjin Medical University, Tianjin, China

**Keywords:** anisometropia, binocular vision, cross-sectional study, intermittent exotropia, near stereoacuity

## Abstract

**Purpose:**

To investigate clinical factors associated with impaired near stereoacuity in children and adolescents with intermittent exotropia (IXT).

**Methods:**

In this hospital-based cross-sectional study, 809 patients aged 3–17 years with IXT were recruited at Tianjin Eye Hospital from January 2021 to February 2022. Near stereoacuity was measured using the Titmus stereo test (impaired defined as >100 s of arc). Factors associated with impaired near stereoacuity were first examined using univariate logistic regression, followed by multivariable logistic models with stepwise adjustment for demographic and refractive-related confounders. Exploratory subgroup analyses were conducted to assess potential heterogeneity of associations across clinical subgroups.

**Results:**

Impaired near stereoacuity was observed in 52.0% (421/809) of patients. In the fully adjusted multivariable model, independent risk factors included anisometropia (odds ratio [OR] = 1.68, 95% confidence interval [CI]: 1.12–2.51), longer disease duration (OR = 1.06, 95% CI: 1.01–1.12), and larger deviation angles at near (OR = 1.02, 95% CI: 1.01–1.03) and distance (OR = 1.02, 95% CI: 1.01–1.04). Associations were consistent across sex, age, and refractive status subgroups. Notably, anisometropia showed a stronger association in basic-type IXT (OR = 3.35, 95% CI: 1.77–6.32).

**Conclusion:**

Impaired near stereoacuity affects over half of pediatric patients with IXT and is independently linked to anisometropia, longer disease duration, and greater deviation angles. Routine assessment of near stereoacuity and refractive status is recommended to guide early intervention and management in this population.

## 1 Introduction

Stereopsis, the highest form of binocular vision, enables depth perception by integrating binocular disparity cues. Intermittent exotropia (IXT), one of the most prevalent forms of childhood-onset strabismus, is characterized by intermittent outward deviation of the eyes while preserving periods of orthotropia (Liu et al., 2023; Ma and Scheiman, 2024). In Asian populations, particularly among Chinese children, IXT prevalence ranges from 3.4 to 3.9% (Wang et al., 2021; Zhang et al., 2021; Wu and Wang, 2024; Chandramouli et al., 2025).

Given the intermittent disruption of binocular alignment in IXT, near stereoacuity serves as a key clinical measure of the resulting binocular sensory function, owing to its ease of assessment and relevance to near-visual tasks in daily life (Liu et al., 2023). However, the factors contributing to impaired near stereoacuity in child and adolescent patients with IXT remain incompletely elucidated. Although some studies have suggested that most patients with IXT are able to maintain normal near stereoacuity (Kang and Lee, 2015; Mohney et al., 2019; Choi et al., 2022), other investigations have reported that a proportion of patients exhibit varying degrees of near stereoacuity impairment, even at an early stage of the disease (Hatt and Gnanaraj, 2013; Pang et al., 2021; Xi et al., 2023; Liu et al., 2023).

Previous research has implicated clinical factors such as disease duration and deviation angle in stereopsis outcomes (Eshaghi et al., 2021; Singh et al., 2022; Wang et al., 2022). However, these studies often relied on univariate analyses, failing to account for inter-variable correlations and potential confounders. Anisometropia, which disrupts the balance of binocular visual input, has also been associated with reduced stereopsis, even in the absence of amblyopia or manifest strabismus (Elamurugan et al., 2022; Potluri et al., 2022). To date, no large-scale study has systematically examined the independent and combined influences of age, disease duration, deviation magnitude, refractive status, and IXT subtype on near stereoacuity in children and adolescents.

This hospital-based cross-sectional study aimed to identify independent clinical factors associated with impaired near stereoacuity in a large child and adolescent IXT cohort and to explore potential heterogeneity across subgroups defined by age, sex, refractive status, and IXT subtype. These findings may guide more comprehensive sensory assessment and tailored management strategies for children and adolescents with IXT.

## 2 Materials and methods

### 2.1 Study design and participants

This hospital-based cross-sectional study consecutively recruited children and adolescents with intermittent exotropia (IXT) who presented to the Department of Pediatric Ophthalmology and Strabismus at Tianjin Eye Hospital between January 2021 and February 2022. Of 954 initially included patients, 94 were excluded due to age outside the target range (3–17 years old) and 51 due to incomplete key data (primarily due to poor cooperation during near stereoacuity or refractive measurements). A total of 809 participants were included in the final analysis.

The study was conducted in accordance with the Declaration of Helsinki and approved by the Ethics Committee of Tianjin Eye Hospital (approval number: KY-2021019). Written informed consent was obtained from the parents or legal guardians of all participants. The study was designed and reported in accordance with the Strengthening the Reporting of Observational Studies in Epidemiology (STROBE) guidelines.

Inclusion criteria were: (1) a deviation angle ≥15 prism diopters (PD) at distance (6 m) or near (33 cm), measured using the prism and alternate cover test (PACT); (2) age-appropriate visual acuity with best-corrected visual acuity (BCVA) of 20/25 or better in both eyes, with no significant interocular difference and no identifiable risk factors for amblyopia; for some younger participants who were less cooperative with visual acuity testing with the standard logarithmic visual acuity chart but were able to complete stereoacuity testing reliably, fixation preference testing revealed central, steady, and maintained fixation in either eye individually, with no objection to monocular occlusion and no identifiable amblyogenic risk factors; (3) no prior strabismus surgery; and (4) sufficient cooperation for all examinations required for analysis.

Exclusion criteria were: (1) restricted ocular motility, dissociated vertical deviation, or nystagmus; (2) vertical deviation ≥5 PD; (3) coexisting ocular pathology or systemic disorders that could affect binocular vision; and (4) poor cooperation or inadequate understanding during stereoacuity or refractive testing.

### 2.2 Data collection and ophthalmic examinations

All examinations were performed by experienced ophthalmologists and optometrists. Refractive errors were fully corrected with spectacles or trial lenses during testing when indicated. Parents or legal guardians completed a standardized questionnaire regarding perinatal history, parental age at childbirth, parental educational level, prior refractive correction, and family history of strabismus.

Deviation angles at near (33 cm) and distance (6 m) were measured using the PACT following 30-min monocular occlusion to suspend fusional mechanisms. IXT subtype was classified according to the difference between near and distance deviation angles as follows: basic type [difference <10 prism diopter (PD)], convergence insufficiency type (near deviation exceeding distance deviation by ≥10 PD), and divergence excess type (distance deviation exceeding near deviation by ≥10 PD) (Burian and Franceschetti, 1970).

All participants underwent cycloplegic refraction. Refractive error was converted to spherical equivalent (SE) using the formula: SE = sphere + ½ cylinder. Anisometropia was defined as an interocular SE difference ≥1.0 diopter (D) (Robaei et al., 2007; Zhou et al., 2023). Refractive status was categorized as myopia (SE ≤ −0.50 D), emmetropia (−0.50 D < SE < +2.00 D), or hyperopia (SE ≥ +2.00 D) (Galvis et al., 2021; Monika and Durajczyk, 2023). When refractive status differed between eyes, the eye with the larger absolute SE value was used to represent the participant’s refractive status.

Near stereoacuity was assessed using the Titmus Stereo Test (Stereo Optical Co., Inc.) at a viewing distance of 40 cm with full optical correction (if required) under standard indoor illumination. Stereoacuity thresholds were recorded in arcseconds (arcsec) and categorized as normal (≤100 arcsec) or impaired (>100 arcsec) (Xu et al., 2022).

Disease duration was determined from parent/guardian report as the time elapsed since first noticed onset of IXT manifestations.

### 2.3 Statistical analysis

All statistical analyses were performed using SPSS software (version 26.0). Continuous variables were tested for normality using the Shapiro–Wilk test. Normally distributed variables are reported as mean ± standard deviation (SD) and were compared using independent-samples *t* tests, whereas non-normally distributed variables are presented as median (interquartile range, IQR) and were compared using the Mann–Whitney *U* test. Categorical variables are presented as frequencies and percentages and were compared using the *χ*^2^ test.

Near stereoacuity status (normal vs. impaired) was treated as the dependent variable. Potential correlates were initially screened by univariate logistic regression. Variables with *p* < 0.10 or deemed clinically relevant were entered into sequential multivariable logistic regression models with progressive adjustment for confounders. Results are reported as odds ratios (ORs) with 95% confidence intervals (CIs).

Exploratory stratified analyses were performed according to sex, age group, refractive status, and IXT subtype. These analyses were descriptive in nature, and no formal interaction tests were conducted.

## 3 Results

### 3.1 Clinical characteristics and distribution of near stereoacuity status

A total of 809 children and adolescents with IXT were included in the analysis, comprising 406 males (50.2%) and 403 females (49.8%). The mean age was 7.4 ± 3.2 years, and the mean age at onset was 5.3 ± 3.3 years. Among the participants, 371 (45.9%) were only children, 347 (42.9%) were delivered vaginally, and 17.6% had anisometropia.

The median (IQR) SE was 0.0 (−1.9, 0.8) D in the right eye and 0.0 (−1.5, 0.8) D in the left eye, with a median interocular SE difference of 0.2 (0.0, 0.8) D. The mean deviation angle was 32.7 ± 10.3 PD at near and 26.1 ± 10.0 PD at distance. By IXT subtype, 443 patients (54.8%) were classified as basic type, 357 (44.1%) as convergence insufficiency type, and 9 (1.1%) as divergence excess type.

Based on near stereoacuity status, 388 patients (48.0%) had normal near stereoacuity, whereas 421 (52.0%) exhibited impaired near stereoacuity. The median disease duration among patients with impaired near stereoacuity was 2.4 (1.5, 3.5) years. Compared with those with normal near stereoacuity, patients with impaired near stereoacuity had a significantly higher prevalence of anisometropia (20.9% vs. 13.9%, *p* = 0.009) and larger deviation angles at distance (27.2 ± 11.2 PD vs. 24.8 ± 8.3 PD, *p* < 0.001) and at near (33.7 ± 11.7 PD vs. 31.6 ± 8.6 PD, *p* = 0.004) (Supplementary Table S1).

### 3.2 Univariate analysis: longer disease duration, anisometropia, and larger deviation angles are associated with impaired near stereoacuity

Univariate logistic regression revealed several clinical factors associated with impaired near stereoacuity (Supplementary Table S2). Participants with anisometropia had higher odds of impaired near stereoacuity than those without anisometropia (OR = 1.63, 95% CI: 1.13–2.37, *p* = 0.009). Each additional year of disease duration increased the odds by approximately 6% (OR = 1.06, 95% CI: 1.01–1.12, *p* = 0.045).

In addition, larger deviation angles were significantly associated with impaired near stereoacuity. For each 1 PD increase in deviation angle, the odds of near stereoacuity impairment increased by approximately 2% at near (OR = 1.02, 95% CI: 1.01–1.03, *p* = 0.005) and 3% at distance (OR = 1.03, 95% CI: 1.01–1.04, *p* = 0.001).

### 3.3 Multivariable analysis: disease duration, anisometropia, and deviation angles are independently associated with impaired near stereoacuity

Multivariable logistic regression analyses were performed using three sequential models: an unadjusted model (Model 1); a model adjusted for age, sex, age at onset, family history of strabismus, mode of delivery, and spectacle wear (Model 2); and a fully adjusted model additionally including refractive status, spherical equivalent, anisometropia, disease duration, and deviation angles at near and distance (Model 3) (Supplementary Table S3).

In the fully adjusted model, anisometropia remained independently associated with impaired near stereoacuity (OR = 1.68, 95% CI: 1.12–2.51, *p* = 0.012). Each additional year of disease duration was associated with a 6% increase in the odds of near stereoacuity impairment (OR = 1.06, 95% CI: 1.01–1.12, *p* = 0.037). Larger deviation angles at near (OR = 1.02, 95% CI: 1.01–1.03, *p* = 0.007) and at distance (OR = 1.02, 95% CI: 1.01–1.04, *p* = 0.002) were also independently associated with impaired near stereoacuity.

### 3.4 Exploratory stratified analysis: the association between anisometropia and impaired near stereoacuity is more pronounced in basic-type IXT

**Figure 1:**
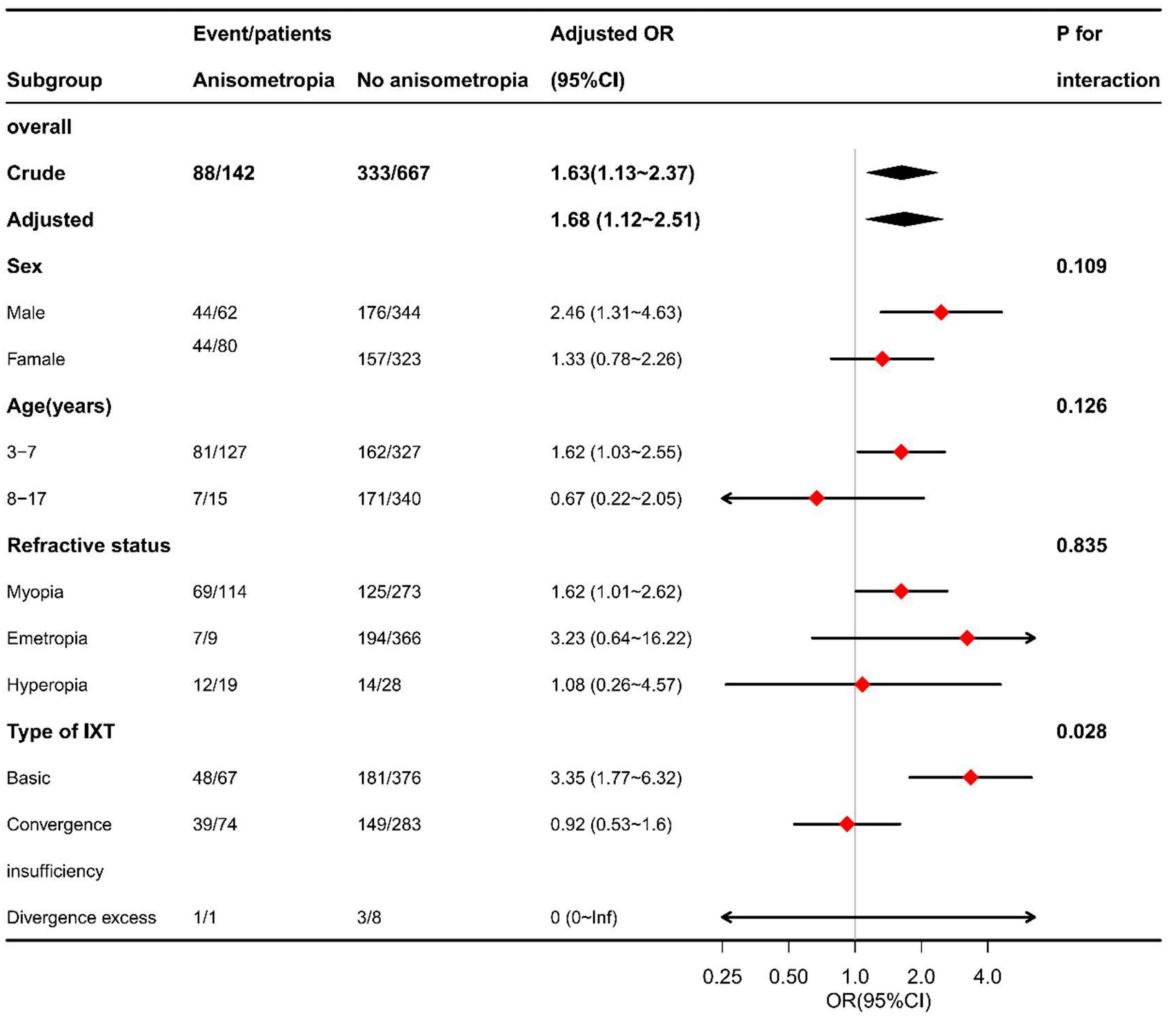
Stratified analyses of the association between anisometropia and impaired near stereoacuity in children and adolescents with intermittent exotropia. Odds ratios (ORs) and 95% confidence intervals (CIs) were derived from the fully adjusted multivariable logistic regression model (Model 3). Stratified analyses were performed according to sex, age group, refractive status, and intermittent exotropia subtype. ORs > 1 indicate a higher likelihood of impaired near stereoacuity associated with anisometropia. IXT, intermittent exotropia; OR, odds ratio; CI, confidence interval.

Based on the fully adjusted model, exploratory stratified analyses were conducted to further examine the association between anisometropia and impaired near stereoacuity. When stratified by sex, age group, and refractive status, the direction of the association remained consistent, with broadly similar effect sizes across subgroups and no apparent heterogeneity (Figure 1).

In contrast, stratification by IXT subtype suggested differential association. Anisometropia was significantly associated with impaired near stereoacuity in patients with basic-type IXT (OR = 3.35, 95% CI: 1.77–6.32), whereas no significant association was observed in patients with convergence insufficiency type IXT (OR = 0.92, 95% CI: 0.53–1.60). Because the number of patients with divergence excess type IXT was small (*n* = 9), the effect estimates in this subgroup were unstable and statistical power was insufficient to detect potential associations. As no formal interaction tests were performed, these findings should be interpreted as exploratory and descriptive.

## 4 Discussion

This large cross-sectional study of 809 children and adolescents with IXT revealed that 52.0% had impaired near stereoacuity, highlighting the frequent compromise of binocular sensory function despite the intermittent nature of the misalignment. Independent predictors of impairment included anisometropia, longer disease duration, and larger deviation angles at both near and distance. Notably, the adverse effect of anisometropia was substantially stronger in basic-type IXT than in convergence insufficiency type.

Normal development of stereopsis depends on clear, symmetric binocular visual input and stable ocular alignment. In IXT, recurrent ocular misalignment and impaired fusion may disrupt stereoscopic function to varying degrees (Hatt et al., 2011; Liebermann et al., 2012; Yam et al., 2013). While a previous study reported poor near stereoacuity in 59.1% of 137 patients (Lee et al., 2014), our study substantiates these findings within a substantially larger cohort of 809 pediatric patients. We identified impaired near stereoacuity in approximately half of the population, providing robust evidence for the high prevalence of sensory deficits in IXT, underscoring the clinical importance of near stereoacuity assessment alongside motor measures such as deviation magnitude and control. Importantly, Titmus Stereo Test only requires relatively simple perceptual judgments, making it suitable for most children aged 3 and more years with appropriate instruction. It should be noted that developmental factors and cognitive ability may influence performance of stereopsis testing. To minimize this potential influence, we only included participants who were able to reliably complete all examinations required for analysis. Children with poor cooperation or inadequate understanding during stereoacuity or refractive testing were excluded in the final analysis. Furthermore, age was explicitly considered in the statistical analysis. Age was included as a covariate in the multivariable regression models, and exploratory stratified analyses by age group showed generally consistent associations between anisometropia, disease duration, deviation magnitude, and impaired near stereoacuity across age strata. These findings suggest that the observed associations were unlikely to be primarily driven by developmental or cognitive differences. It is also important to note that although a BCVA of 20/25 in some participants is below optimal visual acuity (20/20), it still exceeds the lower limit of normal for children of visual developmental age (20/40 for children aged 3–5 years and 20/30 for those aged ≥6 years). Therefore, while we acknowledge that subtle lower acuity compared with optimal visual acuity may influence stereoacuity, the potential impact in this cohort is limited given the generally age-appropriate and balanced visual acuity across participants.

The association between disease duration and stereopsis in IXT remains controversial. Several longitudinal studies have reported low rates of stereoacuity deterioration over short- to medium-term follow-up in untreated children (Holmes et al., 2011; Mohney et al., 2019), and disease duration alone has been considered a poor predictor of stereopsis impairment (Zhao et al., 2024). Nevertheless, the present cross-sectional analysis demonstrated an independent association between longer disease duration and impaired near stereoacuity. This finding does not contradict previous longitudinal evidence but suggests that, across disease stages, stereoscopic function may vary substantially, and disease duration may serve as a clinically relevant temporal indicator associated with sensory outcomes.

Deviation magnitude has also been implicated in stereopsis impairment in IXT. Prior studies have reported associations between near deviation angle and stereoscopic function (Superstein et al., 2017). Extending these observations, our results indicate that both near and distance deviation angles are independently associated with impaired near stereoacuity, suggesting that greater ocular misalignment may compromise effective binocular fusion and depth perception.

Anisometropia emerged as an independent risk factor, consistent with its known disruption of balanced binocular input and subsequent reduction in stereopsis (Elamurugan et al., 2022; Potluri et al., 2022). Previous studies have shown that anisometropia is associated with reduced stereopsis even in the absence of strabismus or amblyopia (Armarnik et al., 2022; Khan et al., 2022). In a population-based study, Robaei et al. reported markedly lower rates of normal stereopsis in children with uncorrected anisometropia compared with emmetropic peers (Robaei et al., 2007). In the present study, which focused on children with IXT and excluded amblyopia, anisometropia remained independently associated with impaired near stereoacuity, highlighting its contribution to stereoscopic dysfunction in this population.

Mechanistic studies suggest that anisometropia-related suppression and expansion of suppression zones may underlie reduced stereoscopic sensitivity (Brooks et al., 1996; Hozumi et al., 2023; Hashemi et al., 2024). In line with these observations, our findings indicate that clinical management of IXT should not focus exclusively on ocular alignment, but also consider timely identification and correction of anisometropia to support binocular sensory function.

Notably, exploratory stratified analyses suggested that the association between anisometropia and impaired near stereoacuity may differ across IXT subtypes. The association was more pronounced in patients with basic-type IXT, whereas no statistically significant association was observed in those with convergence insufficiency type IXT. These findings raise the possibility that IXT subtype could act as an effect modifier in the relationship between anisometropia and stereoscopic impairment. Clinically, reduced fusional capacity in convergence insufficiency type IXT may represent a dominant contributor to stereoacuity impairment, potentially attenuating the independent contribution of refractive asymmetry. In contrast, patients with basic-type IXT may retain relatively greater fusion potential, such that anisometropia-related binocular imbalance is more readily reflected in stereoacuity outcomes. It should be noted that basic-type IXT constituted the majority of our cohort, the overall conclusions of this study are primarily driven by findings in this subtype. In contrast, the small number of patients with divergence excess type IXT precluded meaningful statistical inference, and the absence of significant associations in this subgroup should not be interpreted as evidence of no effect.

Several limitations should be acknowledged. The cross-sectional design precludes causal inference. Residual developmental or cognitive influences cannot be completely excluded, which is an inherent limitation of cross-sectional pediatric studies. Residual confounding related to unmeasured fusional function cannot be completely excluded despite multivariable adjustment. Particularly, quantitative fusion parameters (such as fusional vergence ranges, Newcastle control scores, etc.) were not consistently available for all participants, as the collection of quantitative fusion measures was not part of the protocol for this primarily observational cross-sectional study. Future prospective studies incorporating standardized quantitative fusion assessments would help further elucidate the mechanisms underlying stereoacuity impairment in IXT. Additionally, the hospital-based single-center design may introduce selection bias and limit generalizability, particularly to other settings and ethnic groups.

## 5 Conclusion

This large cross-sectional study demonstrates a high prevalence of impaired near stereoacuity in children and adolescents with IXT. Longer disease duration, anisometropia, and larger deviation angles at both near and distance were independently associated with increased risk of near stereoacuity impairment. These findings support a comprehensive clinical approach that integrates sensory assessment and refractive evaluation alongside ocular alignment and fusion parameter measures, and they highlight the need for future large-scale prospective studies to clarify mechanisms and guide intervention strategies.

## Data Availability

The original contributions presented in the study are included in the article/supplementary material, further inquiries can be directed to the corresponding author.
